# Spatial quasi-bound states of Dirac electrons in graphene monolayer

**DOI:** 10.1038/s41598-024-53329-0

**Published:** 2024-02-15

**Authors:** Mohammed Miniya, Outmane Oubram, Abdel Ghafour El Hachimi, Luis Manuel Gaggero-Sager

**Affiliations:** 1 Centro de Investigación en Ingenierías y Ciencias Aplicadas, Universidad Autónoma Del Estado de Morelos, Av. Universidad 1001, Col. Chamilpa, 62209 Cuernavaca, Morelos Mexico; 2 Facultad de Ciencias Químicas e Ingeniería, Universidad Autónoma Del Estado de Morelos, Av. Universidad 1001, Col. Chamilpa, 62209 Cuernavaca, Morelos Mexico; 3 Departamento de Química, Centro de Investigación en Síntesis Química (CISQ), Universidad de la Rioja, Complejo Científico Tecnológico, 26004 Logroño, Spain

**Keywords:** Electronic properties and materials, Semiconductors, Graphene, Two-dimensional materials

## Abstract

Our study investigated the emergence of spatial quasi-bound states (QBSs) in graphene monolayers induced by rectangular potential barriers. By solving the time-independent Dirac equation and using the transfer matrix formalism, we calculated the resonance energies and identify the QBSs based on probability density functions (PDF). We analyzed two types of structures: single and double barriers, and we find that the QBSs are located within the barrier region, at energies higher than the single barrier. Additionally, we observe QBSs in the double barrier and their position depends on the distance and width of the well between the two barriers. The width and height of the barrier significantly impact the QBSs while the well width influences the resonance energy levels of the QBSs in the double barrier. Interestingly, the QBSs can be manipulated in the graphene system, offering potential for optoelectronic devices. Finally, our results demonstrated that the spatial localization of these states is counter-intuitive and holds great promise for future research in optolectronic devices.

Graphene consists of a single monolayer of carbon atoms. It has attracted a lot of theoretical and experimental attention^1,2^. One factor that makes graphene highly attractive is that the electrons in graphene behave like chiral massless fermions^3^, described by a two-dimensional Dirac equation. In graphene, Dirac electrons, by means of Klein tunneling can penetrate through high and wide potential barriers^4,5^. As a result, controlling Dirac electrons by means of electrical potential is considered a very difficult task^6–8^. However, the electron penetration into the potential barrier is reduced if the propagation is at certain angles^9^.

The peculiarity of the graphene spectrum, namely the existence of degeneration points, makes the local density of carriers very sensitive to the electric field^10^. This paves the way for the creation of localized electronic states close to the zero energy of the two-dimensional Dirac Hamiltonian^10–12^.

In the usual way, Dirac electrons in graphene cannot be effectively confined to a finite spatial area^13^. This effect is disadvantageous for the creation of useful structures in graphene-like structures. However, using electrostatic potentials, some theoretical attempts have been suggested to trap Dirac electrons in graphene to form quasi-bound states (QBSs).

In the continuous spectrum all states are accessible and all are not square integrable^14–16^. However sometimes in very specific situations, there are states that behave almost like bound states^14–18^. This duality, which appears only in very special situations and at certain values, which is called quasi-bound states^18–21^.

According to some works in the literature, QBS are bounded states within a rather short lifetime (trapping time). In contrast, bounded states can be defined as those in which the carriers are absolutely bounded within an infinite time^22–25^.

Later, it has been shown that sharp circular potential wells in graphene display QBSs and the electron is trapped for a limited amount of time before escaping down through the Klein tunnelling^26–28^, the transport resonance in Z-shaped graphene nanoribbons (GNR) has been studied.

The results indicate that the QBSs induce resonant transmission of electrons around the Dirac point. QBSs are mainly confined in the zigzag edges of the GNR. In addition, their energies and lifetimes depend on the structural size^29–31^. Additionally, the QBSs in graphene quantum dots (QDs) has been investigated. The existence of QBSs by applying an external parabolic potential to a graphene strip was reported^32–34^. Moreover, cylindrical symmetric potentials were investigated in single and bilayer graphene QDs. It was demonstrated that in bilayer graphene, rather narrow QBSs appear when the energy is smaller than the barrier height. The broadening of states in the graphene bilayer increases as the orbital momentum becomes larger, which is in contrast with the case of the graphene monolayer^35^.

This study used the solution of the Dirac equation and transfer matrix formalism, along with the continuity of the wave function, to determine the transmission coefficient and resonance energies. Probability density functions are calculated for each resonance energy to plot the QBSs in the case of one or two barriers (see Figs. 1, 2). The results demonstrated that Dirac electrons are localized as QBSs above the barrier and are influenced by the barrier’s width and height. Furthermore, we investigated the QBSs in the case of two rectangular barriers with identical height and width. Our findings reveal that the well width between the two barriers has a significant impact on QBS control.

**Figure 1:**
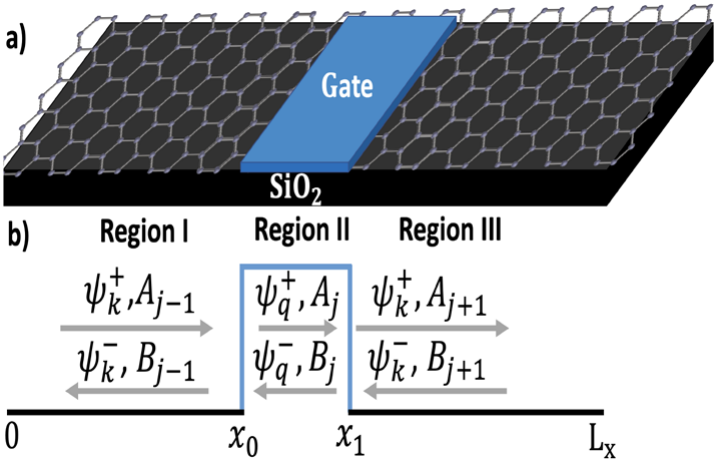
(**a**) Schematic illustration of a rectangular electrostatic barrier on a structure-based graphene monolayer. Regions I, III are wells and II is a barrier. (**b**) The potential profile indicates the incident and reflected particles in each region. $W_{B}$ is the barrier width, $V_{0}$ is the barrier height, and $L_{x}$ is the length of the structure.

**Figure 2:**
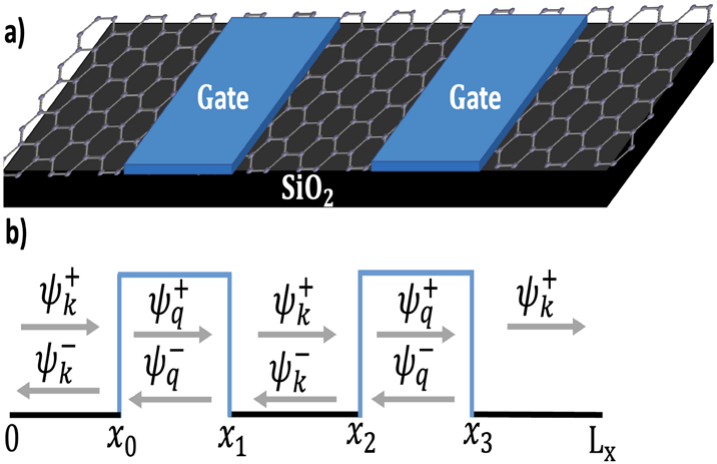
(**a**) Schematic illustration of two rectangular electrostatic barriers on a structure-based graphene monolayer. (**b**) The potential profile indicates the incident and reflected particles in each region. $d_{s}$ is well width.

We consider the potential represented by the following equation:

1 $$\begin{aligned} V(x)= {\left\{ \begin{array}{ll} 0, &{} if \, \, x \, \in Region \, I\\ V_{0}, &{} if \, x \, \in Region \, II \\ 0,&{} if \, x \, \in Region \, III \end{array}\right. }. \end{aligned}$$

Under the effect of the potential, the Dirac cone of graphene monolayer moves proportionately to the applied voltage $V_{0}$^36^.

The Dirac equation is used to study the graphene monolayer-based structure.

2 $$\begin{aligned} {[} v_{F} ( \vec { \sigma }\cdot \vec {p} )+ V_{0}] \psi (x,y) = E \psi (x,y), \end{aligned}$$

where $\sigma _{i}$ with $i=x,y,z$ is the Pauli matrix, $v_{F}$ is the Fermi velocity and $V_{0}$ is the applied potential, and $\overrightarrow{p}$ is the momentum.

By solving (Eq. 2), we can obtain the eigenfunctions and eigenvalues. Usually, the electronic wave functions in graphene are described by two-components (i.e: pseudo-spins). The two components correspond to the quantum mechanical amplitudes of finding the particle in the well-barrier. The function $\psi ^{\pm }_{q_{j}}(x,y)$ represents the wave function in the barrier region (Region II, Fig. 1), namely:

3 $$\begin{aligned} \psi ^{\pm }_{q}(x,y)=\dfrac{1}{\sqrt{2}} \begin{pmatrix} 1 \\ v^{\pm } \end{pmatrix} e^{\pm i q_{x}x + i k_{y} y}, \end{aligned}$$

where $q_{x},k_{y}$ are the wave vector and $v^{\pm }$ are the wave function components given by:

4 $$\begin{aligned} v^{\pm }=\dfrac{\hbar v_{F} (\pm q_{x} + i q_{y})}{E-V_{0}}, \end{aligned}$$

with the corresponding dispersion relation:

5 $$\begin{aligned} E=\pm \hbar v_{F} \sqrt{q^{2}_{x}+k^{2}_{y}}+V_{0}. \end{aligned}$$

In the case of the well regions (Region I, III), the wave function is given as:

6 $$\begin{aligned} \psi ^{\pm }_{k}(x,y)=\dfrac{1}{\sqrt{2}} \begin{pmatrix} 1 \\ u^{\pm } \end{pmatrix} e^{\pm i k_{x}x + i k_{y} y}, \end{aligned}$$

where $k_{x},k_{y}$ are the wave vector and $u^{\pm }$ are the corresponding wave function components given by:

7 $$\begin{aligned} u^{\pm }= \pm sign(E) e^{\pm i \theta }, \end{aligned}$$

with dispersion relation:

8 $$\begin{aligned} E= \pm \hbar v_{F} \sqrt{k^{2}_{x}+k^{2}_{y}}. \end{aligned}$$

To determine the amplitudes of the wave functions in each well-barrier, the solution of the Dirac equation is written in matrix form using the continuity conditions as a function of the amplitudes:

9 $$\begin{aligned} \begin{pmatrix} A_{j} \\ B_{j} \end{pmatrix} = D_{j}^{-1}P_{j}(x_{j})^{-1}D_{j-1}P_{j-1}(x_{j})\begin{pmatrix} A_{j-1} \\ B_{j-1} \end{pmatrix}, \end{aligned}$$

where $D_{j}$, $P_{j}(x_{j})$ are the dynamic matrix and propagation matrix, respectively, and are defined as:

10 $$\begin{aligned} D_{j} = \begin{pmatrix} 1 &{} 1 \\ u_{j}^{+} &{} u_{j}^{-} \end{pmatrix}, P_{j}(x_{j}) = \begin{pmatrix} e^{-q_{j}x_{j}} &{} 0 \\ 0 &{} e^{+q_{j}x_{j}} \end{pmatrix}, \end{aligned}$$

where $j=1,2,\ldots ,n$. If *j* is odd, then the component of the wave function $u_{j}=u_{\pm }$ and the wave vector will be equal to $q_{j}= k_{x}$ that correspond to the well region (Region I). If *j* is even, then $u_{j}=v_{\pm }$ and $q_{j}=q_{x}$ that correspond to barrier region (Region II).

The transfer matrix^37^ can be calculated as:

11 $$\begin{aligned}{} & {} \begin{pmatrix} A_{0} \\ B_{0} \end{pmatrix} = D_{0}^{-1} \left[ \prod _{j=1}^{n} D_{j} P_{j} D_{j}^{-1}\right] D_{0} \begin{pmatrix} A_{n+1} \\ B_{n+1} \end{pmatrix}, \end{aligned}$$

12 $$\begin{aligned}{} & {} \begin{pmatrix} A_{0} \\ B_{0} \end{pmatrix} = M \begin{pmatrix} A_{n+1} \\ B_{n+1} \end{pmatrix}, \end{aligned}$$

where $M(E, \theta )$ given by:

13 $$\begin{aligned} M(E,\theta ) = \begin{pmatrix} M_{11} &{} M_{12} \\ M_{21} &{} M_{22} \end{pmatrix}, \end{aligned}$$

The coefficients can be determined $A_{j-1}$, $B_{j-1}$, $A_{j}$, $B_{j}$, $A_{j+1}$, $B_{j+1}$, using the continuity condition and transfer-matrix approach. We assume that $A_{j-1} = 1$ and $B_{j+1}=0$, where this condition, reflects that the wave function can only be transmitted through the barrier. The probability density function (PDF) that corresponds to each region, in terms of amplitude coefficient, is given by:

In region (I):

14 $$\begin{aligned} \mid \psi _{I}(x) \mid ^{2}= \bigg | D_{0} P_{0}(x) \begin{pmatrix} A_{0} \\ B_{0} \end{pmatrix} \bigg |^{2}. \end{aligned}$$

In region (II):

15 $$\begin{aligned} \mid \psi _{II}(x) \mid ^{2} =\bigg | D_{1} P_{1}(x) \begin{pmatrix} A_{1} \\ B_{1} \end{pmatrix} \bigg |^{2}. \end{aligned}$$

In region (III):

16 $$\begin{aligned} \mid \psi _{III}(x)\mid ^{2} =\bigg | D_{2} P_{2}(x) \begin{pmatrix} A_{2} \\ B_{2} \end{pmatrix}\bigg |^{2}. \end{aligned}$$

In *n*-region:

17 $$\begin{aligned} \mid \psi _{n}(x) \mid ^{2} =\bigg | D_{n} P_{n}(x) \begin{pmatrix} A_{n} \\ B_{n} \end{pmatrix}\bigg |^{2}. \end{aligned}$$

All probability density functions are normalized to the whole interval domain of the structure. Once we have the transfer matrix, we can calculate the transmission coefficient using the following equation:

18 $$\begin{aligned} T(E, \theta )= \left| \dfrac{A_{trans}}{A_{incid}} \right| ^{2}=\dfrac{1}{\left| M_{11} \right| ^{2}}, \end{aligned}$$

where $M_{11}$ is the first element of the transfer matrix.

**Figure 3:**
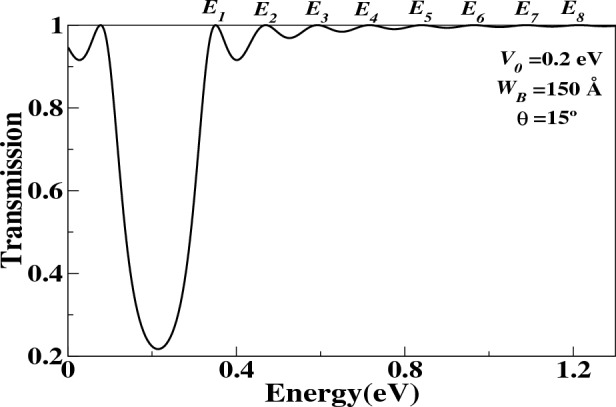
The transmission probability as a function of the Fermi energy. $(E_{i})$ is the resonance energy where is $i=1,2,\ldots ,N=8$. $\theta =15^{\circ }$ is the angle incident of Dirac electron.

**Figure 4:**
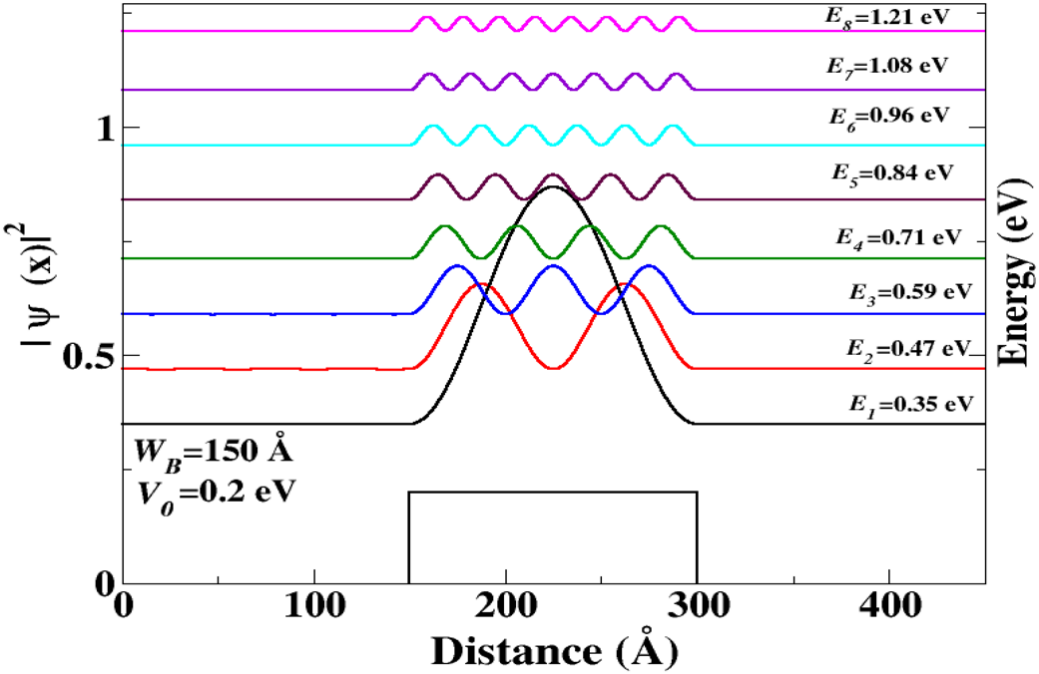
The probability density function (PDF) as a function of the distance *x*. QBSs corresponding to the energies resonance levels $E_{i}$. The resonance energies of the Dirac electron are chosen such that the transmission coefficient is $T = 1$.

Initially, we investigated the presence of QBSs in a single barrier (SB) structure. To achieve this, we calculated the probability density function (PDF) for each resonance energy by utilizing the transmittance. Figure 3 displays the transmission probability as a function of the Fermi energy. The parameters used in this structure include the barrier height ($V_{0} = 0.2$ eV), the length of the barrier ($W_B = 150$ Å), and the incidence angle of the Dirac electron ($\theta = 15^{\circ }$). These parameters were selected to obtain the QBSs energies of the graphene system in the linear approximation regime. We analyzed the QBSs at different resonance energies obtained from the transmission curve for $T = 1$.

Figure 4 illustrates the resonance states and probability of QBSs existence for various resonance energies. Figure 4 exhibits the scenario where the barrier width is $W_B=150$ Å. The curves show that the first energy level $E_1$ corresponds to the initial QBSs and is manifested above the energy barrier. At the succeeding energy level, $E_2$, distinctive characteristics of the QBSs were observed.

The behavior of QBSs changes from one energy level to another, as the number of nodes increases with energy. This means that the quasi-bound states appear in series above the barrier, with their spatial distributions having a maximum of one, two, three, and so on, as seen in the case of bound states in conventional semiconductors^37^.

To investigate the evolution of different QBSs, we explored other resonance energies, and the results are displayed in Fig. 4. At higher energy levels, the QBSs exhibit the same characteristic, with the number of maxima and minima increasing as the resonance energy increases, similar to what was previously observed in Fig. 4.

**Figure 5:**
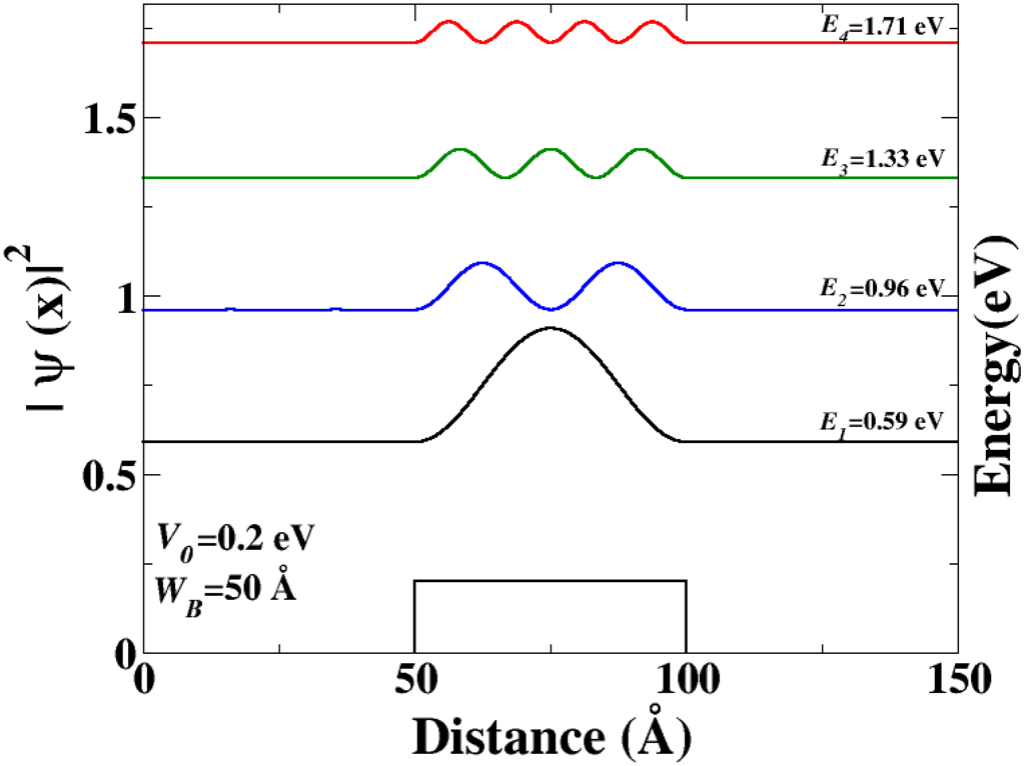
The PDF as a function of the distance *x*. $E_{1}$, $E_{2}$, $E_{3}$, and $E_{4}$ correspond to the resonance energies of QBS above the barrier, respectively. The energies of the Dirac electron are chosen such that the transmission coefficient $T = 1$. The length of the barrier is $W_{B} = 50$ Å, and the electron incident angle is $\theta = 15^{\circ }$.

We also investigated the effect of barrier width on QBSs. QBSs for a barrier width of $W_{B}=50$ Å, were determined, and the results are shown in Fig. 5. The Dirac electrons are quasi-localized at an energy level above the barrier. We found that the QBS energies for the barrier with width $W_{B}=50$ Å, are higher than those of the barrier with width $W_{B}$=150 Å. Furthermore, the energies of the QBSs decrease as the width of the barrier increases, which is consistent with the dispersion relation expression given in Eq. (5).

**Figure 6:**
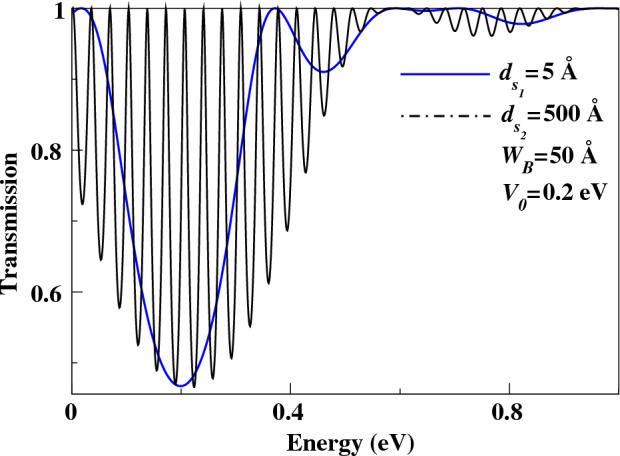
The transmission probability as a function of the Fermi energy. The transmission coefficient is calculated for two well widths, for instance, $d_{s_{1}}$, $d_{s_{2}}$. The electron incident angle is $\theta =15^{\circ }$.

It is noteworthy that the quasi-bound states (QBSs) situated within the energy barrier can be modulated by both the width ($W_{B}$) and the height ($V_{0}$) of the barrier. Therefore, by varying the barrier width in this system, different QBSs can be probed. In this study, we have examined various barrier widths and found that the resonance energies are affected by changes in $W_{B}$.

**Figure 7:**
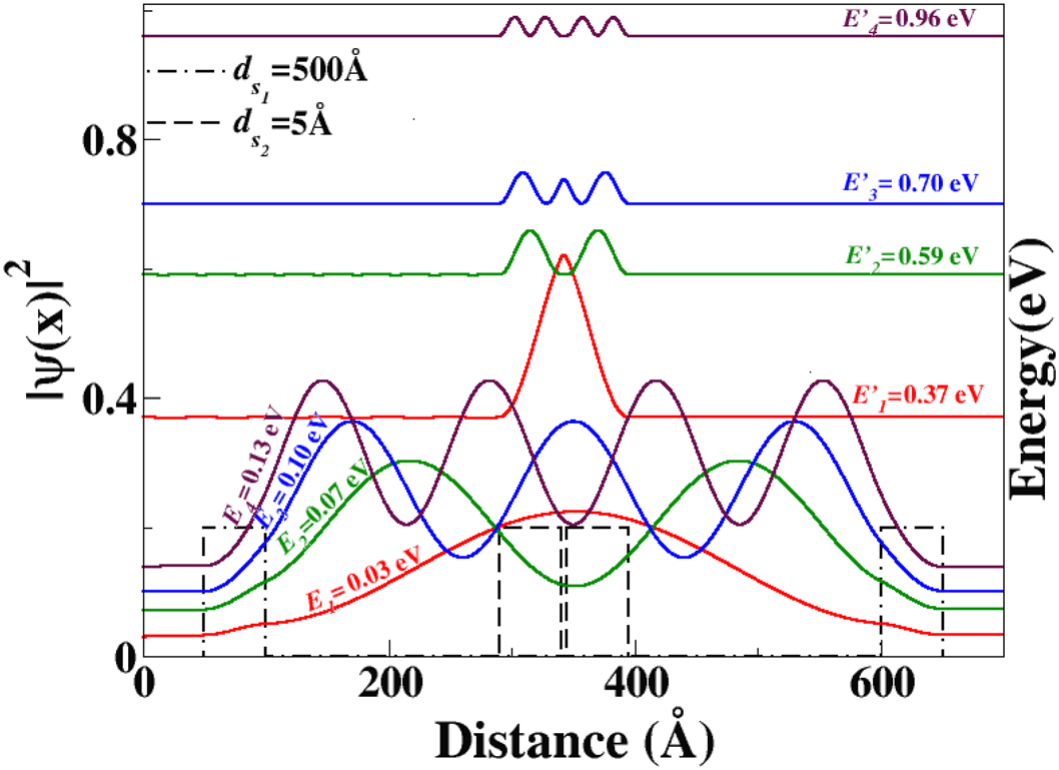
The PDF is a function of the distance *x*. ($E_{1}^{'}, E_{2}^{'}, E_{3}^{'}, E_{4}^{'}$), ($E_{1}, E_{2}, E_{3}, E_{4}$), are the energies of resonance corresponding to the QBSs in the case of $d_{s_{1}}$=5 Å  and $d_{s_{2}}$=500 Å, respectively. The barrier height is $V_{0}=0.2 \,$ eV, and the electron incident angle is $\theta =15^{\circ }$.

Likewise, our investigation into the impact of barrier height on the quasi-bound states (QBSs) revealed that there was no noticeable effect on the energy levels. In contrast, varying the angle of incidence resulted in gradual changes in the energy levels of the QBSs. Specifically, increasing the angle of incidence had a discernible impact on the QBSs.

We have investigated the impact of barrier width ($W_{B}$) and height ($V_{0}$) on the energy difference between quasi-localized states, $\Delta E = E_{i+1} - E_{i}$, of the quasi-bound states (QBSs). Our findings suggest that varying $W_{B}$ does not lead to any noticeable change in $\Delta E$, whereas altering $V_{0}$ results in an increase in $\Delta E''$ proportionate to the increase in barrier height. Furthermore, our study on the effect of different angles of incidence indicates that the energy difference, $\Delta E$, remains unaffected by changes in the incidence angle. Therefore, we report no dependence of the energy difference on the angle of incidence of Dirac electrons.

An empirical formula has been derived for the energy difference between quasi-localized states, $\Delta E$, which is dependent on the width ($W_{B}$) of the energy barrier. The formula is expressed as follows:

19 $$\begin{aligned} \tilde{\Delta _{s}}= & {} (W_{B}-150)(W_{B}-300)*1.48.10^{-5}-(W_{B}-50)(W_{B}-300)*1.6.10^{-5}\nonumber \\{} & {} +(W_{B}-50)(W_{B}-150))*1.6.10^{-6} \end{aligned}$$

Where $\tilde{\Delta _{s}}$ is the difference energy in eV.

It is noteworthy that the behavior of the quasi-bound states (QBSs) in a single energy barrier is remarkable. Specifically, all of the first states are physically located within the barrier region, and their probability distribution is concentrated in that region. Furthermore, it has been observed that the energy difference between successive levels of the QBSs, $\Delta E$, is generally constant at approximately 0.12 eV.

**Figure 8:**
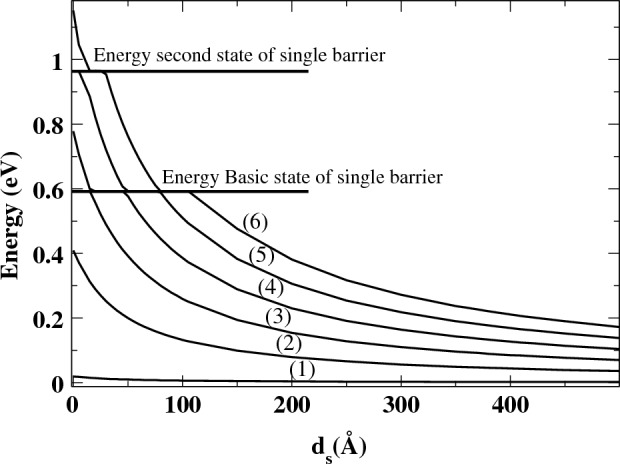
Energies resonance of QBSs as a function of well distance $d_{s}$. The curves (1) to (6) indicate, respectively, the energy levels of QBSs.

This paper presents another study conducted to investigate the presence of quasi-bound states (QBSs) and the impact of a double barrier (DB) structure, as well as the effect of well width ($d_{s}$). Specifically, we examined the well width between two barriers for various systems (see Fig. 2). Figure 6 illustrates the transmission coefficient as a function of Fermi energy for a double barrier (DB) structure. Specifically, we investigated two cases: one with a well width of $d_{s_{1}}=5$ Å, (blue curve) and another with a well width of $d_{s_{2}}=500$ Å, (black curve). Our findings indicate that the transmission probability of the second case exhibits a higher number of resonance peaks, whereas a smoother transmission curve is observed for the first case. Thus, it can be concluded that the resonance energies are significantly influenced by the well distance ($d_{s}$), as demonstrated in Fig. 6. Consequently, a considerable impact on the quasi-bound states (QBSs) is observed.

Figure 7 displays the resonance energies of QBSs as a function of the well width ($d_s$) between two barriers. The energy levels of QBSs are significantly affected by the well distance, as shown in the figure. Specifically, as the well width $d_s$ increases, the energy levels of QBSs decrease. This result confirms the previous findings presented in the paper. It is important to note that the well width plays a crucial role in controlling the QBSs and their corresponding resonance energies.

Furthermore, it is observed that as the well distance ($d_{s}$) increases, the energy levels decrease, indicating that the QBSs can be tuned accordingly. Additionally, the energy level of the QBSs can also be modified by the incidence angle. Consequently, we examined various angles of incidence for both cases, i.e., $(d_{s} = 5$ Å) and $(d_{s} = 500$ Å). Our findings indicate that in both cases, the energy levels are dependent on the angle of incidence, while the energy difference between two successive levels ($\Delta E$) remains constant. As a result, it can be concluded that $\Delta E$ does not exhibit any dependence on the angle of incidence.

The results depicted in Fig. 8 support our previous findings illustrated in Fig. 7. As mentioned earlier, the energy levels decrease as the well width ($d_{s}$) increases. It is worth noting that for values of $d_{s}$ that are close to zero, the QBSs in the DB system exhibit similar characteristics to those observed in the SB case. It is worth noting that certain states of the SB with specific $d_{s}$ can be maintained and also appear in the case of another barrier width, which is an important feature. For instance, at energy $E = 0.59$ eV, the QBSs are located in the third state of the SB with $W_{B} = 150$ Å, (as shown in Fig. 4). Interestingly, the last state also appears in the case of $W_{B} = 50$ Å, and its energy level becomes the ground state of this barrier width (as shown in Fig. 5). Furthermore, it is possible that some states of the SB case coexist in the DB, which is another surprising characteristic.

In this study, we explored the quasi-bound states (QBSs) in single and double rectangular potentials using the transfer matrix formalism. We calculated the resonance energies and the probability density function to determine the spatial location of the QBSs. We found that the QBSs are located above the barrier and can be tuned by adjusting the width and height of the barrier. Interestingly, the QBSs also appeared in the case of double barriers and their energy levels were found to be affected by the width of the well between the barriers. The study is unique in that it calculated the wave function and established the spatial region where these states are located. The investigation of QBSs in graphene-based low-dimensional systems also revealed new characteristics. Finally, this study provides insights into the behavior of QBSs and their dependence on different parameters in the system.

## Data Availability

The data that support the findings of this study are available from the corresponding authors upon reasonable request.
